# PaO_2_/FiO_2_ ratio forecasts COVID-19 patients’ outcome regardless of age: a cross-sectional, monocentric study

**DOI:** 10.1007/s11739-021-02840-7

**Published:** 2021-10-12

**Authors:** Gaia Sinatti, Silvano Junior Santini, Giovanni Tarantino, Giovanna Picchi, Benedetta Cosimini, Francesca Ranfone, Nicolò Casano, Maria Antonella Zingaropoli, Nerio Iapadre, Simone Bianconi, Antonietta Armiento, Paolo Carducci, Maria Rosa Ciardi, Claudio Maria Mastroianni, Alessandro Grimaldi, Clara Balsano

**Affiliations:** 1 School of Emergency and Urgency Medicine, Department of Clinical Medicine, Life, Health and Environmental Sciences-MESVA, University of L’Aquila, L’Aquila, Italy; 2 Clinical Medicine and Surgery Department, Federico II University Medical School of Naples, Naples, Italy; 3 Department of Infectious Disease, San Salvatore Hospital, L’Aquila, Italy; 4 Department of Pneumology, San Salvatore Hospital, L’Aquila, Italy; 5 Department of Public Health and Infectious Diseases, Sapienza University of Rome, Rome, Italy; 6 Emergency and Acceptance Department, San Pietro Hospital, Rome, Italy; 7 Department of Internal Medicine and Public Health, University of L’Aquila, L’Aquila, Italy

**Keywords:** COVID-19, SARS-CoV-2, Pandemic, PaO_2_/FiO_2_ ratio, Atypical pneumonia

## Abstract

We studied the predictive value of the PaO_2_/FiO_2_ ratio for classifying COVID-19-positive patients who will develop severe clinical outcomes. One hundred fifty patients were recruited and categorized into two distinct populations (“A” and “B”), according to the indications given by the World Health Organization. Patients belonging the population “A” presented with mild disease not requiring oxygen support, whereas population “B” presented with a severe disease needing oxygen support. The AUC curve of PaO_2_/FiO_2_ in the discovery cohort was 0.838 (95% CI 0.771–0.908). The optimal cut-off value for distinguishing population “A” from the “B” one, calculated by Youden’s index, with sensitivity of 71.79% and specificity 85.25%, LR+4.866, LR−0.339, was < 274 mmHg. The AUC in the validation cohort of 170 patients overlapped the previous one, i.e., 0.826 (95% CI 0.760–0.891). PaO_2_/FiO_2_ ratio < 274 mmHg was a good predictive index test to forecast the development of a severe respiratory failure in SARS-CoV-2-infected patients. Moreover, our work highlights that PaO_2_/FiO_2_ ratio, compared to inflammatory scores (hs-CRP, NLR, PLR and LDH) indicated to be useful in clinical managements, results to be the most reliable parameter to identify patients who require closer respiratory monitoring and more aggressive supportive therapies. Clinical trial registration: Prognostic Score in COVID-19, prot. NCT04780373 https://clinicaltrials.gov/ct2/show/NCT04780373 (retrospectively registered).

**Supplementary Information:**

The online version contains supplementary material available at 10.1007/s11739-021-02840-7.

## Introduction

The natural history of SARS-CoV-2 infection is extremely variable, ranging from asymptomatic or mild infection to a severe acute distress respiratory syndrome (ARDS), characterized by a typical hyperinflammatory response associated with a microangiopathy and a widespread thrombosis [1–4].

In the face of the rapidly spreading disease and the large number of infected people, there is an urgent need to cluster patients in risk categories by identifying reliable parameters related to COVID-19 disease progression to stratify high-risk patients, who will suffer rapid disease progression to severe complications and death.

The clinical respiratory symptoms of SARS-CoV-2 infection frequently do not correspond to the severity of lung damage; therefore, to correctly stratify COVID-19 patients, it is important to evaluate the acute lung injury based on well-accepted test for acute lung injury and ARDS, such as the partial pressure of arterial oxygen (PaO_2_) to fraction of inspired oxygen (FiO_2_) ratio (PaO_2_/FiO_2_) [5–8].

Several inflammatory biomarker abnormalities have been identified to stratify COVID-19 patients who will develop a severe disease [9–11]. High-sensitivity C-reactive protein (Hs-CRP) as well as neutrophil (NEU)-to-lymphocyte (LYM) ratio (NLR) and platelet (PLT)-to-lymphocyte (LYM) ratio (PLR) have been investigated in SARS-CoV-2 infection and have been indicated to be useful in clinical managements of infected patients [12, 13]. Moreover, lactate dehydrogenase (LDH), an intracellular enzyme that well correlates with lung damage and inflammation, has been associated with worse outcomes in patients with viral infections and has been identified as an independent indicator for predicting severity and mortality in patients with COVID-19 [14, 15].

The aim of our study was to assess the PaO_2_/FiO_2_ ratio as a reliable prognostic biomarker to forecast the progression of COVID-19 patients towards the most severe form, according to WHO criteria, comparing its predictive value with other canonical inflammatory biomarkers.

## Materials and methods

### Study design

We conducted a cross-sectional monocentric study, adhering to the principles of the Declaration of Helsinki. The study was approved by the institutional ethic committee (License Prot. n. 71726) and informed consent was obtained from participating patients at the San Salvatore Hospital, L’Aquila, Italy.

### Discovery cohort

#### Inclusion criteria

During the first and second pandemic “wave” of SARS-CoV-2 epidemic, from April 15, 2020, to November 31, 2020, 153 patients were recruited at the Department of Infectious Disease and at the Department of Pneumology of the San Salvatore Hospital, L’Aquila, Italy. All patients showed positivity to RT-PCR assay from nasopharyngeal swab sample and clear futures to chest CT. At the arrival patients were consecutively enrolled and biochemical parameters were collected at three time points: (1) the day of hospitalization due to the respiratory symptoms onset (T0); (2) day 3 (T3) and day 7 (T7) after the admission. At the same time points, arterial blood gas was performed to evaluate the respiratory function based on partial pressure of arterial oxygen (PaO_2_) to fraction of inspired oxygen (FiO_2_) ratio (PaO_2_/FiO_2_ ratio).

Out of the 153 patients, three were drop-outs. Specifically, one of them refuses to keep on participating, and two were suspected to have a neoplastic disease. Of the remaining 150 patients, 94, who presented with mild COVID-related symptoms, were assigned to population “A” and 56 with severe COVID-19-related symptoms to population “B”, according to the global clinical status. In particular, patients were divided in two reference populations using the term “A” or “B”, if the evaluation of the WHO score at T7, was between 1 and 4 or between 5 and 8, respectively. The fundamental criterion to be part of the population “A” or “B” was the need or not of oxygen support by the infected patients.

In particular, the World Health Organization (WHO) was engaged to classify the severity of this infection for COVID-19 trial endpoints (http://www.who.int/blueprint/priority-diseases/key-action/novel-coronavirus/en/). The WHO ordered the severity of the infectious disease in “low” (WHO score 1–2) that correspond to the presence of mild symptoms, “moderate” for patients who no required supplemental oxygen (WHO scores 3–4), and “severe” for all patients who needed oxygen support (WHO scores 5–8 high-flow oxygen requirement continuous positive airway pressure (CPAP), non-invasive ventilation (NIV), multi-organ support and death).

#### Exclusion criteria

Patients were excluded if they were known to be pregnant, had a history of vasculitis or connective tissue disease and were subjected to long-term corticosteroids or chronically immunosuppressed, received antivirals, anti-interleukin (IL)-1, anti-IL-6 or anti-TNF therapy, or underwent dialysis for chronic kidney disease or had suspect/active neoplasia.

### Predictive index test

As predictive index test was used the PaO_2_/FiO_2_ ratio, the most reliable diagnostic criteria for acute lung injury and ARDS [16].

### Principal outcome

As principal outcome, we wondered to investigate if PaO_2_/FiO_2_ ratio was reliable for classifying SARS-CoV-2-infected patients who will show a severe disease.

### Secondary outcome

We were interested in comparing the predictive value of PaO_2_/FiO_2_ ratio to inflammatory scores (hs-CRP, NLR, PLR and LDH), already known to be useful in predicting the outcome of SARS-CoV-2-infected patients.

### Validation cohort

To validate PAO_2_/FiO_2_ ratio as a reliable COVID-19 risk biomarker for the identification of patients who will develop critical illness, according to the WHO criteria, we retrospectively analyzed the clinical and biochemical data of 170 patients (broken into Population “A” and “B”) collected by two hospitals of central Italy. The variables required for validating PaO_2_/FiO_2_ ratio were collected and cross-checked by two experienced physicians (Table S2).

The number of patients, both in the discovery and in the validation cohort, who were given oxygen support is shown in Table S3.

### Statistical evaluation

Variables were not normally distributed and were expressed as median plus 1st and 3rd interquartile, after controlling by the Shapiro–Wilk test. Difference in medians was evaluated by the Mann–Whitney *U* test. Frequencies were analyzed by the chi-square.

As measure of association, we chose to study predictions that were carried out by two types of regression techniques.

Dealing with the binary dependent variable or dichotomous outcome, i.e., Population “A”/“B” at time point T7, and one independent variable the simple logistic regression was used, calculating the odds ratio, the Std. Err. the *z*, the *P* and 95% confidence intervals (CI).

In case of more than one independent variable, the multiple logistic regression was performed, always calculating the same afore mentioned parameters, by using the enter method alone. This choice was based on having strong hypotheses about which variables belong in the model. There were implanted three models, i.e., model A comprehending all the inflammatory parameters, model B adjusted for PaO_2_/FiO_2_ ratio and model C, further adjusted for age.

ROC analysis was used as a diagnostic decision-making. Indicatively, to measure the performance of the binary classification test (index test) was studied the best cut-off, coupled with the sensitivity, specificity, positive likelihood ratio (LR +) = sens/(1-spec) and the negative likelihood ratio (LR-) = (1-sens)/spec), pointing out that the more the LR + is > 1, the more likely the outcome. On the contrary, the more a likelihood ratio for a negative test is < 1, the less likely the outcome.

Furthermore, the correct classification percentage of Population “A”/“B” at the time point T7 and the area under the receiver operating characteristic (AUROC/AUC) were performed to evaluate the most appropriate models (the highest specificity and sensitivity), under the nonparametric assumption. The cut-off (cut-points) with the highest specificity and sensitivity was calculated by the means of Youden’s Index according to [17].

## Results

### Dynamic profile of SARS-CoV-2-infected patients

Of the whole cohort of patients 38% were female, the mean age was 61 (ranging from 51.75 to 70.25). Among all the parameters analyzed the following displayed statistically significant changes during the first week of hospitalization: PaO_2_/FiO_2_ ratio, HR, procalcitonin, D-dimer, INR, ALT, AST, gamma-GT, FiO_2_, hs-CRP, platelet and lymphocyte count (Table 1).

**Table 1:** Demographic variable symptoms and biochemical parameters in SARS-CoV-2 at T0–T3 and T7

Characteristics	All cohort T0 (N.150)	All cohort T3 (N.150)	All cohort T7 (N.142)
Age (years)	61.00 (51.75–70.25)		61.00 (52.00–70.00)
Female	57 (38%)		54 (38%)
Male	93 (62%)		88 (62%)
Fever	129 (86.00%)	31 (20.94%) (148)	9 (7.30%) (123)
RR (breaths/min)	18 (16–22)	18 (15–20)	17 (15–18)
HR (bpm/min)	80.00 (72.75–94.25)	78.50 (70.00–85.00)*	73.00 (67.00–80.00)***^,##^
Procalcitonin (ng/mL)	0.0860 (0.0310–0.2275)	0.1810 (0.0633–0.5508)*	0.0310 (0.0200–0.1450)^##^
Hs-CRP (mg/dL)	5.370 (1.754–10.360)	3.485 (1.040–10.300)	1.510 (0.430–4.280)***^,###^
Ferritin (ng/mL)	508.8 (302.6–1010.0)	794.4 (211.7–1528.8)	753.0 (323.5–1055.6)
Fibrinogen (mg/dL)	532.0 (415.5–609.5)	485.0 (397.0–608.0)	532.5 (377.5–610.3)
D‐dimer (mg/L)	0.68 (0.42–1.29)	1.07 (0.53–1.94)*	0.63 (0.43–0.83)^#^
INR	1.085 (1.030–1.160)	1.125 (1.068–1.242)*	1.125 (1.080–1.210)*
ALT (UI/mL)	25.50 (15.25–37.75)	37.00 (20.00–69.00)**	36.00 (23.00–67.50)**
AST (UI/mL)	28.00 (21.00–38.75)	34.50 (27.00–46.50)*	29.00 (23.00–40.00)
Gamma-GT (UI/mL)	39.00 (21.00–66.00)	59.50 (35.25–88.00)*	53.50 (35.75–82.75)*
ALP (UI/mL)	56.00 (46.00–68.50)	52.00 (44.00–61.00)	56.00 (40.00–70.00)
Creatinine (mg/dL)	0.830 (0.740–0.995)	0.780 (0.680–0.870)	0.800 (0.705–0.965)
PaO_2_ mmHg	68.00 (58.30–78.85)	65.00 (56.00–76.6)	67.50 (58.10–74.88)
FiO_2_ mmHg	0.21 (0.21–0.28)	0.34 (0.21–0.60)***	0.28 (0.21–0.60)***
PaO_2_/FIO_2_ mmHg	300.0 (207.0–352.0)	192.5 (110.0–300.0)***	246.0 (117.4–322.0)**
LDH (UI/L)	293 (231–371)	308 (244–424)	256 (226–335)
NLR	4.135 (2.484–7.985)	4.981 (2.295–9.564)	3.767 (2.170–9.202)
PLR	197.6 (142.1–288.6)	181.1 (106.4–281.1)	205.5 (125.2–323.0)
Neutrophil (10^9^/L)	4.32 (2.83–6.56)	5.03 (2.86–7.77)	5.34 (3.16–7.70)
Platelet (10^9^/L)	196.0 (154.5–254.0)	227.0 (146.5–294.8)	261.0 (194.0–309)***
Lymphocyte (10^9^/L)	0.970 (0.660–1.335)	0.920 (0.613–1.503)	1.230 (0.755–1.895)*^,#^

Values are reported as medians and first-third quartiles for the continuous variables and percentages for the categorical variables. *vs T0; # vs T3. Statistical significance was assessed by *p* value (*P*) thresholds: *or # *P* < 0.05; **or ## *P* < 0.01; *** or ### *P* < 0.001, (Kruskal–Wallis test)

ALT (T0 vs T3, P < 0.01 and T3 vs T7, *P* < 0.01), AST (T0 vs T3, *P* < 0.01) and gamma-GT (T0 vs T3, *P* < 0.05 and T3 vs T7, *P* < 0.05) were significantly increased in our cohort of patients during the progression of the infection.

Among the respiratory function parameters, FiO_2_ and PaO_2_/FiO_2_ ratio displayed significant negative trend from T0 to T3 (*P* < 0.001 and *P* < 0.001, respectively), that appeared to be significantly improved at day 7 (*P* < 0.001 and *P* < 0.05, respectively).

Platelet and lymphocyte count were significantly increased from T3 to T7 (*P* < 0.001 and *P* < 0.05, respectively).

### Characteristics of the whole population and of both cohorts “A” and “B”

The demographics and clinical characteristics are shown in Table 2.

**Table 2:** Demographics variables symptoms and biochemical parameters in Population “A” and “B”

Characteristic	All cohort (N.150)	Population A (N.94) Non-severe	Population B (N. 56) Severe
Age (years)	61.00 (51.75–70.25)	57.50 (41.75–64.75)	68.00 (62.25–79.75)***
Female	57 (38%)	37 (39.36%)	20 (35.71%)
Male	93 (62%)	57 (60.64%)	36 (64.29%)
Fever	129 (86.00%)	84 (89.36%)	45 (80.00%)
Cough	107 (71.33%)	63 (67.02%)	44 (78.57%)
RR	18 (16–22)	17 (15–19)	21(17–28)***
FC	80.00 (72.75–94.25)	80.00 (72.00–93.25)	83.50 (73.00–99.50)
Comorbidities	97 (64.67%)	36 (38.30%)	33 (58.93%)
Length of stay	12 (7.25–19.00)	8.50 (6.00–13.00)	18.00 (11.25–26.00)***
Procalcitonin (ng/mL)	0.0860 (0.0310–0.2275)	0.0580 (0.0255–0.1220)	0.1390 (0.0618–0.3803)***
Ferritin (ng/mL)	508.8 (302.6–1010.0)	450.0 (239.1–804.7)	1101.0 (711.0–1741.0)**
Fibrinogen (mg/dL)	532.0 (415.5–609.5)	518.0 (381.0–596.0)	580.5 (511.0–569.8)*
D-dimer (mg/L)	0.680 (0.420–1.290)	0.570 (0.380–0.810)	1.180 (0.703–1.930)***
INR	1.085 (1.030–1.160)	1.075 (1.023–1.120)	1.110 (1.043–1.670)**
ALT (UI/mL)	25.50 (15.25–37.75)	22.00 (16.00–37.00)	27.00 (13.50–39.00)
AST (UI/mL)	28.00 (21.00–38.75)	25.00 (21.00–34.00)	33.00 (21.50–49.50)
Gamma-GT (UI/mL)	39.50 (21–66)	38.50 (20.75–55.25)	40.00 (24.50–86.00)
ALP (UI/mL)	56.00 (46.00–68.50)	52.00 (43.00–64.00)	68.50(61.25–85.50)***
Creatinine (mg/dL)	0.830 (0.740–0.995)	0.830 (0.735–0.935)	0.835 (0.74–1.263)
PaO_2_ mmHg	68.00 (58.30–78.85)	70.90 (63.00–81.75)	58.30 (51.10–75.00)**
FiO_2_ mmHg	0.21 (0.21–0.28)	0.21 (0.21–0.21)	0.28 (0.21–0.50)***
PaO_2_/FiO_2_ (mmHg)	300.0 (207.0–352.0)	232.0 (290.0–375.5)	202.0 (118.3–270.0)***
Hs-CRP (mg/dL)	5.370 (1.754–10.360)	3.715 (1.095–7.830)	8.960 (3.530–12.970)***
LDH (UI/L)	293 (231–371)	267.5 (222.3–335.5)	353.0 (279.0–482.0)***
NLR	4.135 (2.484–7.985)	3.370 (2.086–5.939)	7.005 (3.759–12.010)***
PLR	197.6 (142.1–288.6)	189.0 (139.4–280.1)	236.7 (162.4–376.6)
Neutrophil (10^9^/L)	4.32 (2.83–6.56)	3.750 (2.478–5.338)	6.095 (4.010–8.638)***
Platelet (10^9^/L)	196.0 (154.5–254.0)	198.0 (158.0–251.0)	195.5 (150.0–279.0)
Lymphocyte (10^9^/L)	0.970 (0.660–1.335)	1.0750 (0.758–1.385)	0.920 (0.555–1.120)*

Values are reported as medians and first-third quartiles for the continuous variables and percentages for the categorical variables. *vs population “A”; Statistical significance was assessed by *p* value (*P*) thresholds: *P < 0.05; **P < 0.01; ***P < 0.001, (Kruskal–Wallis test)

The rate of severe (population “B”) and moderate cases (population “A”) was 37.4% and 62.6%, respectively.

The population “B” was characterized by the presence of older patients respect to the population “A” (median 57.50 vs 68.00, *P* < 0.001), without any difference between cohorts concerning gender (chi square, *P* = 0.65). Fever (86%) and cough (71.3%) were the first and most common symptoms at admission, and, in this regard, no significant differences between the two populations were observed. RR was higher in the population “B” [21 (17;28) vs 17 (15;19), *P* < 0.05].

The length of stay of the whole cohort of patients was 12 days (7.25–19.00), population A and population B length stay were 8.5 (6.00–13.00) and 18 (11.25–26.00) days, respectively (Table 2).

Most severe cases (population “B”) exhibited higher rate of co-morbidities (58.93 vs 38.30%) without reaching significance. The rate of patients worsening to ARDS was 38.6% and mortality was 9.3%.

Procalcitonin, ferritin, fibrinogen, D‐dimer, international normalized ratio (INR) and alkaline phosphatase (ALP) were significantly higher in the population “B” respect to “A” (*P* < 0.001, *P* < 0.01, *P* < 0.05, *P* < 0.001, *P* < 0.01, *P* < 0.001, respectively), whereas the liver functional parameters were not significantly different between the two populations.

Lymphocytes [median 0.970 (0.660–1.335)], neutrophil [4.32 (2.83–6.56)] and platelet [196.0 (154.5–254.0)] were collected in whole patients and significant differences in neutrophil and platelet count were observed in the population “B” respect to “A” [median 227 (146.5–294.8) and 261 (194–309), *P* < 0.001, respectively].

### PaO_2_/FiO_2_ ratio versus LDH, hs-CRP, NLR and PLR in the population “A” and “B”

Logistic regression analysis revealed a significant negative prediction of the clinical outcome at day 7 by PaO_2_/FiO_2_ ratio (*P* < 0.001).

Similarly, LDH, hs-CRP, and PLR were positively correlated with a poor outcome (*P* < 0.001, *P* < 0.001, *P* = 0.016, respectively) (Table S1).

To evaluate the predictive power of the PaO_2_/FiO_2_ ratio, we performed three models of multivariable regression analysis.

Among the three different models, the model A was performed to correlate the inflammatory parameters (hs-CRP, NLR, PLR and LDH) at the time of hospitalization with the clinical outcome at T7. Our analyses showed that the most representative variables were LDH and hs-CRP (*P* = 0.002 and *P* = 0.039, respectively) (Table 3). Surprisingly, when we adjusted the model for PaO_2_/FiO_2_ ratio (model B, Table 3), we observed a completely different scenario, letting us think that the predictive ability of the respiratory function parameter likely obscures the prognostic value of the inflammation parameters (Table 3). To confirm the result of model B we performed a multivariable regression analysis adjusted for age (model C, Table 3).

**Table 3:** Multivariable logistic regression models for Prediction of population “A” and “B” at days 7

Model A *R*^2^ = 0.1876				
Characteristic	O.R	*z* value	C. I	*P* value
NLR	0.999	− 0.29	0.994–1.004	0.772
PLR	1.002	1.90	1.000–1.005	0.058
Hs-CRP	1.075	**2.07**	1.004–1.151	0.039*
LDH	1.007	**3.05**	1.002–1.011	0.002**

Model B *R*^2^ = 0.3491				
Characteristic	O.R	*z* value	C. I	*P* value
NLR	1.204	**2.05**	1.081- 1.438	0.040*
PLR	1.001	0.93	0.998–1.004	0.355
Hs-CRP	0.978	− 0.52	0.900–1.063	0.604
LDH	1.002	0.77	0.997–1.007	0.438
PaO_2_/FiO_2_	0.985	− **3.89**	0.977–0.922	0.000***

Model C *R*^2^ = 0.4416				
Characteristic	O.R	*z* value	C. I	*P* value
NLR	1.227	**2.23**	1.025–1.468	0.026*
PLR	1.002	1.01	0.998–1.006	0.311
Hs-CRP	0.935	− 1.59	0.861–1.016	0.113
LDH	1.001	0.46	0.995–1.008	0.642
PaO_2_/FiO_2_	0.982	− **3.82**	0.973–0.991	0.000***
age	1.075	**3.51**	1.033–1.120	0.000***

Significant differences are highlighted in bold

Model B was adjusted for PaO_2_/FiO_2_ ratio and Model C was adjusted for age. Statistical significance was assessed by *p* value (*P*) thresholds: *P < 0.05; **P < 0.01; ***P < 0.001

Remarkably, when we analyzed age, at univariate analysis, as a single predictor, it resulted very significant (*P* < 0.001, Table S1).

### Developing a clinical prediction model

**Fig. 1:**
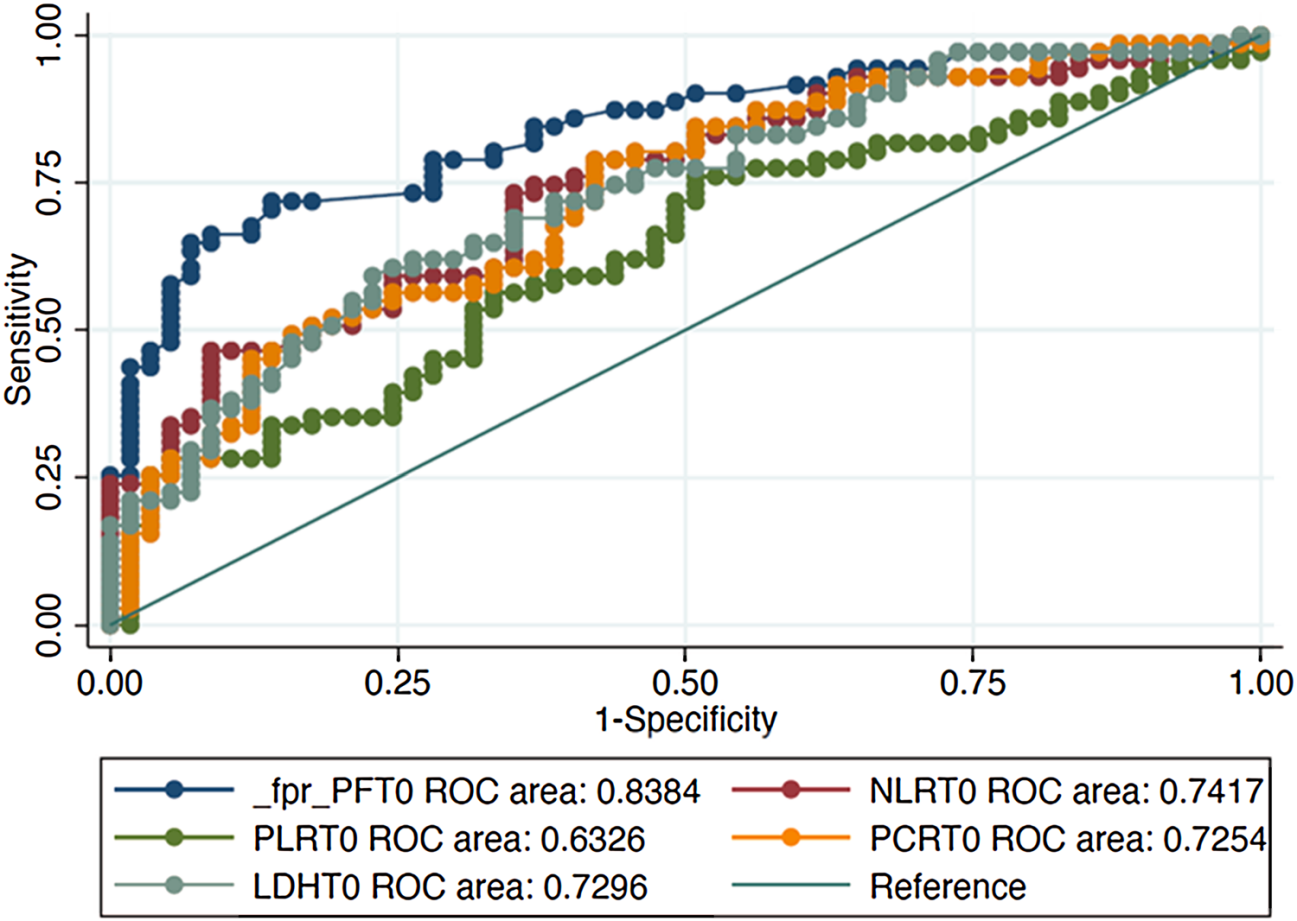
AUC curves of PaO_2_/FiO_2_ ratio, hs-CRP, NLR, PLR and LDH of 150 COVID-19 positive patients

Dealing with the diagnostic accuracy of an index test, we generate the AUC curves of all the studied parameters. AUC curve of PaO_2_/FiO_2_ ratio for differentiating the population “A” and “B”, which was 0.838, (95% CI 0.771–0.908). The PaO_2_/FiO_2_ appeared as the most reliable prognostic biomarker compared to hs-CRP, NLR, PLR and LDH (AUC curves of 0.725, 0.741, 0.632, 0.729, respectively), see Fig. 1.

The optimum PaO_2_/FiO_2_ ratio cut-off value to separate population “A” from “B” was < 274 mmHg, with sensitivity and specificity of 71.79% and 85.25% (LR + 4.8661, LR−0.3309), respectively.

### Model validation

**Fig. 2:**
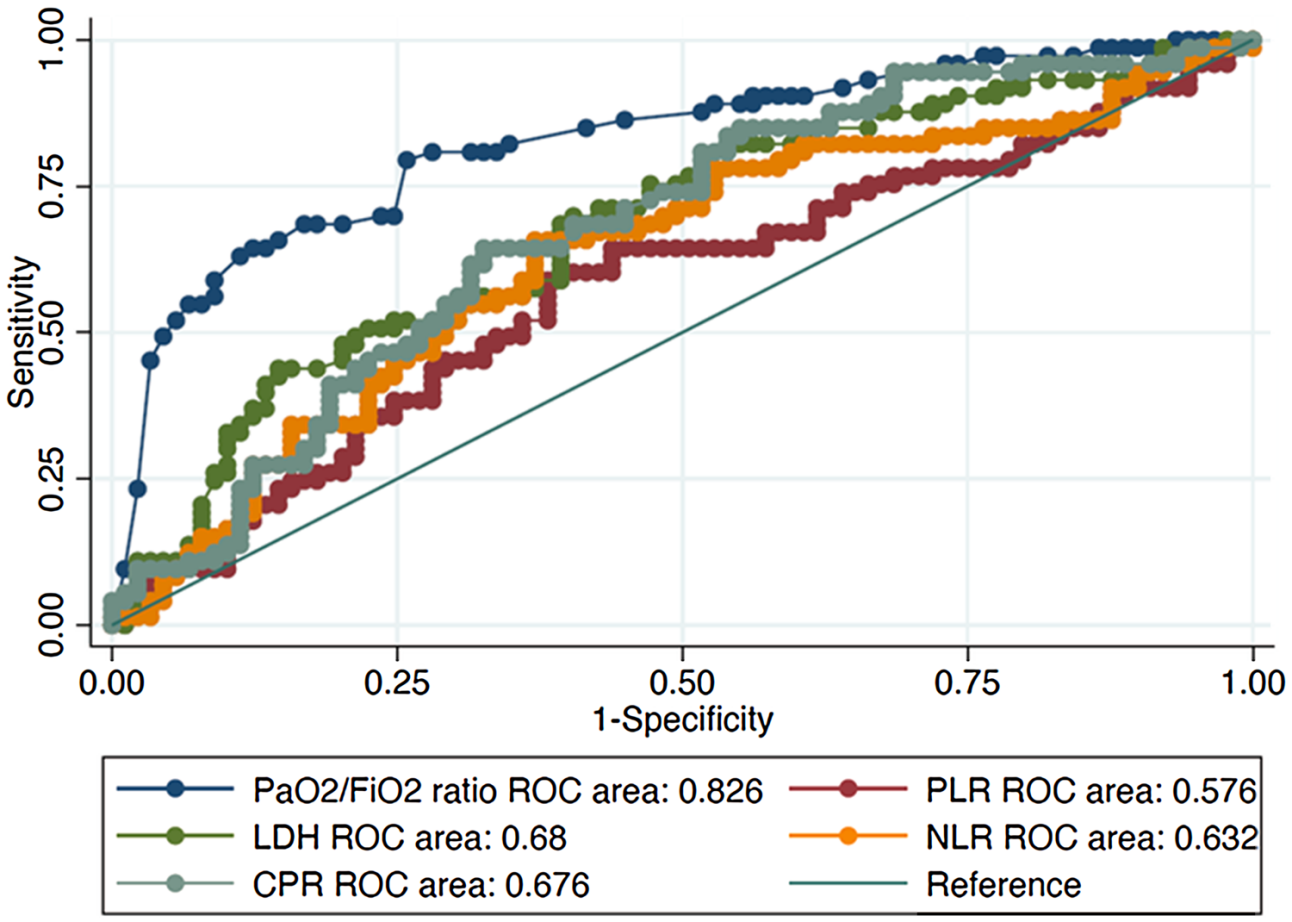
AUC curves of PaO_2_/FiO_2_ ratio, hs-CRP, NLR, PLR and LDH of the validation cohort

To establish its reproducibility, we tested the reliability of the PaO_2_/FiO_2_ ratio in the validation cohort, including retrospectively 170 patients, 100 were female, with a median age of 68.00 years (Table S2). The AUC of the PaO_2_/FiO_2_ ratio resulted to be 0.826 (95% CI 0.760–0.891), Fig. 2. The cut-off neared that was found in the discovery cohort with sensitivity of 82.50% and specificity 71.74%, LR + 2.9192, LR−0.2439.

## Discussion

Summarizing the key results, our research highlights that PaO_2_/FIO_2_ ratio could be considered a reliable prognostic biomarker for patients’ management of COVID-19 pneumonia, as demonstrated by the AUC curve that indicate as cut-off the value < 274 mmHg. The performance of this biomarker was more than satisfactory with a good accuracy based on AUCs, in both the discovery and validation cohorts, of 0.838 and 0.826, respectively.

It is known that COVID-19 prognosis is worse in older patients, but in an interesting way, age did not modify the prediction by PaO_2_/FiO_2_ ratio. Presenting the importance of the study, we think that one of the strengths of our work is that we tracked levels of biomarkers with the aim to establish trends for each patient towards the successive phases of the disease.

The likely contribution of this work to literature consists in evidencing that in our cohort of patients, among the variables analyzed (PaO_2_/FiO_2_ ratio, hs-CRP, NLR, PLR and LDH), PaO_2_/FiO_2_ ratio was the best independent prognostic biomarker for forecasting pneumonia progression toward ARDS in COVID-19 patients.

Describing similar data of literature, our findings were consistent with those of previous studies on LDH, hs-CRP, NLR, PLR [1, 8–12] that associated the afore mentioned prognostic markers with poor disease outcome. However, the reliability of PaO_2_/FiO_2_ ratio in comparison with other inflammatory tests to forecast the worst outcomes of COVID-19-positive patients has not yet been highlighted.

Various indices have been used to describe pneumonia progression towards ARDS, such as the arterial to alveolar O2 difference, the intrapulmonary shunt fraction, the oxygen index and the PaO_2_/FIO_2_ ratio [18]. Of these different indices, the PaO_2_/FIO_2_ ratio, because of its accuracy but also its simplicity, could be taken into serious account also by hospitalists in their every-day practice when dealing with OVID-19.

Concerning this specific pulmonary test, Grasselli et al. in 1591 patients, admitted to an intensive care unit (ICU), found a reduced median of 160 mmHg (IQR 114–220) [19]. According to our results, Santus et al. discovered that a moderate-to-severe impairment in PaO_2_/FiO_2_ ratio was independently associated with a threefold increase in risk of intra-hospital mortality, concluding that the severity of respiratory failure is useful to identify patients at higher risk of mortality [20].

Our study covered a time period during which advances and changes in treatment approaches may have differently reduced the levels of inflammatory biomarkers among patients hospitalized with COVID-19, but this aspect does not affect the importance of our index parameter, on the contrary it reinforces its reliability.

## Conclusion

Could our results fill existing gaps in the field that had not been previously exposed? We aimed to give to clinicians a way to discriminate SARS-CoV-2-positive patients with different fate. We intentionally categorized our cohort in “low” (population “A”) or “high” (population “B”)-risk patients in such a way that clinicians could make decisions quickly based on local or regional conditions and opportune therapeutic approaches. In fact, we consider of great importance the access to good clinical and supportive care for providing a more or less aggressive care whereby the patient’s outcomes might be optimized.

### Limitations

Possible limitations exist in this paper. The study was conducted with a not so large sample size and on data deriving only from the central Italy, which could limit the value of PaO_2_/FiO_2_ ratio as a prognostic biomarker representative of a general population.

Moreover, although the PaO_2_/FiO_2_ ratio is simple and easily available, it has a suboptimal power in categorizing patients with ARDS [21].

### Future directions

As work in progress, we will follow up these cohorts of patients. Nowadays, everlasting effects of COVID-19 are still largely unknown. Long-term pulmonary and systemic complications have been reported in some patients after recovery, with residual fatigue or muscle weakness, chest CT abnormalities, cardiovascular and neurological complications [22, 23].

## Supplementary Information

Below is the link to the electronic supplementary material.

Supplementary file1 (DOCX 15 KB)

Supplementary file2 (DOCX 14 KB)

Supplementary file3 (DOCX 16 KB)

## Data Availability

The datasets used and/or analyzed during the current study are available from the corresponding author on reasonable request.

## Abbreviations

ARDS: Acute distress respiratory syndrome
PaO_2_: Partial pressure of arterial oxygen
FiO_2_: Fraction of inspired oxygen
hs-CRP: High-sensitivity C-reactive protein
NEU: Neutrophil
LYM: Lymphocyte
NLR: Neutrophil-to-lymphocyte ratio
PLT: Platelet
PLR: Platelet-to-lymphocyte ratio
LDH: Lactate dehydrogenase
CPAP: Continuous positive airway pressure
NIV: Non-invasive ventilation
LR: Likelihood ratio
INR: International normalized ratio
ALT: Alanine aminotransferase
AST: Aspartate aminotransferase
ALP: Alkaline phosphatase
GGT: Gamma-glutamyl transferase
HR: Heart rate
RR: Respiratory rate
