# Supplementary material for: PaO2/FiO2 ratio forecasts COVID-19 patients’ outcome regardless of age: a cross-sectional, monocentric study
Source: Intern Emerg Med. 2021 Oct 12;17(3):665–73. doi: 10.1007/s11739-021-02840-7 (PMC8505469; doi:10.1007/s11739-021-02840-7)
Supplement: Supplementary file 1 — Supplementary file1 (DOCX 15 KB) [file 11739_2021_2840_MOESM1_ESM.docx]

**Table S1.** Univariate logistic regression analysis of parameters of population “A” and “B” associated with severe outcome of SARS-CoV-2 infection at day 7. Statistical significance was assessed by p-value (P) thresholds: * P <0.05; ** P <0.01; *** P <0.001.

| Characteristic | O.R. | z-value | C. I. | R2 | P value |
| --- | --- | --- | --- | --- | --- |
| Age | 1.077 | 5.13 | 1.047/1.109 | 0.183 | **0.000***** |
| PaO2/FiO2 | -0.017 | -5.32 | -0239/-0.011 | 0.278 | **0.000***** |
| Hs-CRP | 1.126 | 3.45 | 1.052/1.205 | 0.087 | **0.001***** |
| LDH | 1.008 | 4.00 | 1.004/1.013 | 0.129 | **0.000***** |
| PLR | 1.002 | 2.41 | 1.000/1.004 | 0.041 | **0.016**** |
| NLR | 0.999 | -0.67 | 0.996/1.001 | 0.006 | 0.503 |
