# Supplementary material for: PaO2/FiO2 ratio forecasts COVID-19 patients’ outcome regardless of age: a cross-sectional, monocentric study
Source: Intern Emerg Med. 2021 Oct 12;17(3):665–73. doi: 10.1007/s11739-021-02840-7 (PMC8505469; doi:10.1007/s11739-021-02840-7)
Supplement: Supplementary file 2 — Supplementary file2 (DOCX 14 KB) [file 11739_2021_2840_MOESM2_ESM.docx]

**Table S2.** Clinical parameters of the validation cohort. Values are reported as medians and first-third quartiles for the continuous variables and percentages for the categorical variables.

| Characteristics | All Cohort  (N.170) |
| --- | --- |
| Age (years) | 68.00 (57.00-79.00) |
| Female | 100 (59%) |
| Male | 70 (41%) |
| Ferritin (ng/mL) | 500.00 (250.00-960.00) |
| D-dimer (mg/L) | 0.74 (0.43-1.40) |
| PaO2 mmHg) | 71.00 (61.00-84.00) |
| FiO2 mmHg | 0.21 (0.21-0.33) |
| PaO2/FIO2 mmHg | 319.00 (218.50-385.50) |
| Hs-CRP (mg/dL) | 5.48 (2.02-11.21) |
| LDH (UI/L) | 290.00 (224.00-375.00) |
| NLR | 4.94 (2.80-9.30) |
| PLR | 219.56 (146.80-336.10) |
