# Supplementary material for: PaO2/FiO2 ratio forecasts COVID-19 patients’ outcome regardless of age: a cross-sectional, monocentric study
Source: Intern Emerg Med. 2021 Oct 12;17(3):665–73. doi: 10.1007/s11739-021-02840-7 (PMC8505469; doi:10.1007/s11739-021-02840-7)
Supplement: Supplementary file 3 — Supplementary file3 (DOCX 16 KB) [file 11739_2021_2840_MOESM3_ESM.docx]

|  | **Discovery cohort** | | **Validation cohort** | |
| --- | --- | --- | --- | --- |
| **Oxygen therapy** | **T0** | **T7** | **T0** | **T7** |
| Nasal cannulae | 10 | 16 | 12 | 18 |
| Ventimask | 23 | 21 | 20 | 27 |
| Oxygen masks with reservoir bags | 4 | 10 | 8 | 15 |
| CPAP MASK | 2 | 2 | 1 | 6 |
| CPAP Helmet | 4 | 1 | 2 | 3 |
| NIV PS | 0 | 1 | 0 | 0 |
| Invasive oxygen therapy | 0 | 7 | 0 | 9 |
